# Human Papillomavirus Vaccine Acceptance (HPV-VA) and Vaccine Uptake (HPV-VU): assessing the impact of theory, culture, and trusted sources of information in a Hispanic community

**DOI:** 10.1186/s12889-023-16628-1

**Published:** 2023-09-14

**Authors:** Gabriel Frietze, Margie Padilla, Jacquelin Cordero, Kristin Gosselink, Eva Moya

**Affiliations:** 1 University of Texas at El Paso, 500 University Ave, El Paso, Texas, 79968 USA; 2 School of Public Health, Dept. of Health Promotion and Behavioral Sciences, The University of Texas Health Science Center at Houston (UTHealth), El Paso Campus, 5130 Gateway East Blvd, El Paso, TX 79905 USA; 3 Burrell College of Osteopathic Medicine, 3501 Arrowhead Drive, Las Cruces, NM 88001 USA

**Keywords:** Hispanic adults, Human Papillomavirus, HPV vaccine uptake, HPV vaccine acceptance, Health Belief Model (HBM)

## Abstract

**Background:**

Human papillomavirus (HPV) is the most common sexually transmitted infection and is associated with many types of cancers that disproportionately impact Hispanics. An HPV vaccine is available for individuals ages 9—45 that can prevent up to 90% of HPV-associated cancers. The current study investigates factors associated with accepting the HPV vaccine in a predominately Hispanic community.

**Methods:**

A cross-sectional study design with an online questionnaire was used to collect data from a community sample of adults between the ages 18–65 residing in a U.S./Mexico border city, El Paso, Texas. Theory-based factors (e.g., the Health Belief Model), culture-based factors (e.g., familism), and trusted sources of information were examined as predictors of HPV-vaccine acceptance (HPV-VA) and HPV-vaccine uptake (HPV-VU).

**Results:**

Community members (*N* = 602, *M*_age_ = 34.65, *SD* = 9.79) who were predominately Hispanic (89.4%) and female (79.6%) participated in the study. Linear regression models revealed that HPV-VA was associated with *household size*, *primary language*, *engagement in organizational activities*, *health-related community stigma, government trust,* and the HBM theory-based factors: *perceived benefits, perceived harm,* and *perceived severity.* Logistic regression analyses revealed that HPV-VU was associated with *household size*, *engagement in non-organizational activities*, *HPV trusted sources of information*, and *perceived safety*.

**Conclusions:**

Adequate HPV vaccination uptake among all vaccine-eligible Hispanics is an important step to lessen the HPV-attributed cancer burden. Our hypothesis that theory-based factors would be associated with HPV-VA and HPV-VU was supported. Our findings have implications for designing trusted, theory-based, and culturally sensitive health communications and interventions to promote vaccines in minority underrepresented communities.

## Background

Human papillomavirus (HPV) is the most common sexually transmitted infection (STI) in the U.S. [10]. Most HPV infections clear on their own without adverse health issues; however, persistent high-risk (oncogenic) HPV infections are attributed to 90% of cervical cancers, 91% of anal cancers, 75% of vaginal cancers, 70% of oropharyngeal cancers, 69% of vulva cancers, and 63% of penile cancers [10]. Annually in the U.S., there are about 44,000 new cases of HPV-associated cancers [10]. An HPV vaccine was originally introduced in 2006 and the Advisory Committee on Immunization Practices (ACIP) recommends the HPV vaccine for children (ages 9–17 years) and adults (ages 18–45 years) [39]. Notably, the HPV vaccine can prevent 7 of the 13 oncogenic types that cause 90% of HPV-associated cancers [39].

HPV-associated cancers exhibit disparities among Hispanics, with cervical cancer rates significantly higher in this population among all other ethnicities [10]. El Paso, TX (80% Hispanic), a Medically Underserved Area, reports higher rates of cervical cancers (10.4 per 100,000) versus state (9.2 per 100,000), and the U.S. (8.0 per 100,000), during 2012–2016 [53]. HPV-associated cancers are linked to modifiable risk factors, such as prevention through adequate vaccination; therefore, understanding youth and adult HPV vaccine uptake behaviors is an important step to lessening the cancer burden. An analysis of the National Health and Nutrition Examination Survey (NHANES; cycles 2011–2012, 2013–2014) data showed 18.3% of the U.S. population (2011–2014) ages 18—33 was estimated to have had an uptake of the HPV vaccine (> 1 dose) before the age of 26 (29.2% in women and 6.9% in men; *p* < 0.001). Chaturvedi et al. [11] reported that the proportion of HPV-vaccine uptake among Hispanic males varied significantly compared to non-Hispanic White males (4.6% and 8.7%, respectively). Contrariwise, up-to-date HPV vaccination rates in El Paso County (66%) were estimated to be the highest in Texas and ranked second in the nation among adolescents between 13 – 17 years old (69% females, 63.2% males), demonstrating vaccine acceptance in the region and a potential to reach 80% vaccination goals per Healthy People 2030 among this age group [44]. However, with ACIP recommendation changes and age expansion in 2019, there is less known about vaccination behaviors among vaccine-eligible (ages 18–45 years old) Hispanic adult populations.

Theoretical frameworks such as the Health Belief Model (HBM; [48]) are useful for understanding the complex interplay of factors associated with behaviors such as getting vaccinated. The HBM proposes that a variety of constructs predict engagement in preventive health behaviors. Perceived benefits is a construct in the HBM that can be defined as the belief that a behavior is effective at preventing a threat (e.g., the perception that a vaccine prevents infection). A meta-analysis by Newman et al. [45] revealed that perceived benefits of the HPV vaccine is strongly associated with HPV vaccine acceptance (*r* = 0.51, *p* < 0.001). Perceived severity is another construct in the HBM that can be defined as the belief that the disease is severe to personal health. Newman et al. [45] reported a small correlation between perceived severity of HPV infection and HPV vaccine acceptance (*r* = 0.09, *p* < 0.001). We further investigate theory-based factors (e.g., perceived benefits and perceived severity) to identify correlates of HPV vaccine acceptance (HPV-VA) and HPV vaccine uptake (HPV-VU). In the current study, HPV-VA is defined as intentions to receive the HPV vaccine and HPV-VU is defined as having already received the HPV vaccine.

The association between knowledge about HPV and HPV-VA has been examined in several studies, yielding mixed findings [9, 5, 14, 19, 27]. In one study, knowledge about HPV was associated with increased intentions to accept the HPV vaccine in a sample of predominately Caucasian women [27]. In contrast, Dempsey et al. [14] aimed to increase HPV knowledge in a sample of predominately Caucasian women by providing an HPV information sheet prior to assessing HPV-VA. The authors found that HPV knowledge scores increased significantly in the treatment group compared to the control group (*p* < 0.001); however, no differences emerged between the intervention and control group related to HPV-VA [14]. A study by Frietze et al. ([19]) also did not detect significant associations between knowledge about HPV and HPV-VA in a sample of Hispanic males. Further studies examining the association between knowledge about HPV and HPV-VA are needed to make sense of these conflicting findings.

One of the strongest correlates of HPV-VA and HPV-VU is having recommendations from a healthcare provider (HCP). A meta-analysis by Newman et al. ([45]) investigated HPV-VA in men and reported that HCP recommendations were positively associated with HPV vaccine endorsement (*r* = 0.42, *p* < 0.01). Newman et al. [46] investigated parents’ HPV-VU for their children and found that having a recommendation from a physician was the strongest correlate of HPV-VU (*r* = 0.46, *p* < 0.001). Furthermore, Newman et al. [46] found that parents’ trust in healthcare providers was positively associated with HPV-VU (*r* = 0.46, *p* = 0.026). It is critical to investigate other sources of HPV information (e.g., the World Health Organization) to identify the most trusted sources for recommending the HPV vaccine.

Marlow et al. [37] investigated trust as a predictor of HPV-VA in a predominantly Caucasian sample of mothers and found that the odds of HPV-VA was higher in mothers who reported trusting in the government and doctors (*OR* = 1.35, *p* < 0.001). Similarly, Nan et al. [41] reported that the odds of HPV-VA was higher in individuals who trust in doctors or health professionals (*OR* = 1.30, *p* = 0.001) and government agencies (*OR* = 1.29, *p* < 0.001). Trust is a particularly important construct to investigate in communities of color given the historical legacies (e.g., Tuskegee Trials) and racial disparities that may contribute to inequities in healthcare, including vaccinations. Harrington et al. [21] conducted a review examining the role of trust on HPV-VU in racial and ethnic minorities and reported HPV-VU was lower in communities of color who did not trust government agencies, healthcare providers, or pharmaceutical companies. Moreover, trust in family members and faith-based organizations was higher in minorities than in their White counterparts [21].

Culture is an important factor that warrants further investigation. Culture influences worldviews, language, and health-seeking behaviors and serves as a critical foundation for influencing practice and research. Research suggests that cultures that value the collective benefits of vaccinations report a higher willingness to vaccinate than cultures that value individual benefits [8]. Hofstede [23] explains that collectivist cultures favor family and group solidarity, whereas individualistic cultures favor independence. A strong value in Hispanics of Mexican origin is being family-oriented and it is common that Hispanic households are larger and multigenerational. It is also common that *abuelos(as*) [Spanish for grandpa or grandma] are living in their adult children’s homes. Assessing family-held beliefs about vaccines appears to be critical for understanding how attitudes are shaped in Hispanic families. In a study by Frietze et al. [19], a strong association emerged when examining personal attitudes about vaccines and family attitudes about vaccines (*r* = 0.82, *p* < 0.001). We further explore the associations between personal and family attitudes about vaccines while investigating other culture-based factors that may be unique to Hispanics of Mexican origin including: 1) language, to explore differences between English and Spanish speakers; 2) household size, to explore the impact of larger families; 3) familism, given that Hispanics value family-cohesiveness; 4) religiosity, given that religion is valued in Hispanic culture; 5) community health stigma, to explore the impact of discrimination in one’s own cultural group. Examining culture-based factors that are associated with HPV-VA and HPV-VU is critical for informing the development of culturally sensitive interventions and health messages promoting the HPV vaccine.

The purpose of the current study is to examine the impact of theory-based factors (i.e., the Health Belief Model), culture-based factors that may be unique to Hispanics of Mexican American origin who reside in our community (e.g., language, household size, familism, and religiosity), and trusted sources of information on HPV-VA and HPV-VU in a predominately Hispanic community. We hypothesized that theory-based factors will be associated with HPV-VA and HPV-VU. Specifically, perceived benefits, perceived safety, and perceived severity will be positively associated with HPV-VA and HPV-VU; in contrast, perceived harm will be inversely associated with HPV-VA and HPV-VU. Culture-based factors and trusted sources of information were explored to yield a more comprehensive understanding of the multitude of factors associated with HPV-VA and HPV-VU.

## Methods

### Participants

Six hundred and two adults (*M*_age_ = 34.65, *SD* = 9.79) who identified predominately as female (79.6%) and Hispanic (89.4%) were included in the study. Nineteen participants were excluded from the analysis due to incorrect responses on an attention check item embedded within the online survey. Informed consent was obtained from all subjects in the study. Eligibility requirements consisted of being between the ages 18–65 and residing in El Paso, Texas. All methods were carried out in accordance with relevant guidelines and regulations by the University of Texas at El Paso’s (UTEP) Institution Review Board (IRB). Additionally, all research protocols were approved by UTEP’s IRB. Recruitment and participation of subjects was approved by UTEP’s IRB.

### Measures

#### Basic demographics survey

An 8-item survey assessed basic demographics such as age, gender, ethnicity, primary language spoken at home (English or Spanish), and related information. Sample item: “Please indicate if you are Hispanic or Latino.” Response options included (1) Non-Hispanic, (2) Hispanic/Latinx/Spanish Descent, and (3) Prefer not to answer.

#### Background questionnaire

A 14-item background survey assessed parental status, household size, and items related to parents vaccinating their children.

#### Sexual experience

Four items assessed sexual experience history and experience with sexually transmitted infections. Sample item: “Have you been sexually active at any point in your lifetime (e.g., oral sex, vaginal sex, anal sex)?” Response options included (0) no, (1) yes, and (2) prefer not to answer.

#### HPV knowledge

Fourteen items developed by Katz et al. [28] assessed HPV knowledge. The assessment included prompts such as*:* “Condoms effectively protect against HPV infection.” Response options included (1) True, (0) False, with a composite score created by averaging correct responses for the items and multiplying by 100 to calculate a percentage.

#### Personal and family beliefs about vaccines

Ten items developed by Frietze et al. [19] assessed personal and family beliefs about vaccines. Sample items: “I believe vaccines cause Autism.” “My family had negative feelings about vaccines while I was growing up.” Response options ranged from (1) Strongly disagree, to (5) Strongly agree.

### Attention check

#### Attention

A single item served as an attention check. Sample item: “If you are paying attention, please select "Blue" for the following response.” Response options included: (1) Red, (2) Green, (3) Blue, and (4) Yellow. The research team predetermined that failure to respond correctly to this item results in the exclusion of the subject’s data from analysis.

### Independent variables

#### Perceived benefits

Three items adapted from Brabin et al. [6] assessed the perceived benefits of the HPV vaccine. Sample items: “I believe the HPV vaccine is effective in preventing genital HPV (e.g., vaginal, penile, or anal).” “I believe the HPV vaccine works in preventing genital HPV (e.g., vaginal, penile, or anal).” “I believe if I get the HPV vaccine, I will be less likely to get genital HPV (e.g., vaginal, penile, or anal).” Response options ranged from (1) Strongly disagree, to (5) Strongly agree. Higher scores indicate greater perceived benefits of the HPV vaccine. A composite score was created by averaging the three items. Previous research has demonstrated acceptable reliability (Cronbach’s α = 0.89; [42]). The Cronbach alpha for the three items was equal to 0.88 in the current study.

#### Perceived safety

Three items adapted from Brabin et al., ([6]) assessed the perceived safety of the HPV vaccine. Sample items: “I worry about the short-term side effects of the HPV vaccine.” “I worry that the HPV vaccine might negatively affect my body.” “I worry that the HPV vaccine might have unknown long-term side effects.” Response options ranged from (1) Strongly disagree, to (5) Strongly agree. Response options were reverse coded for all three questions so that higher scores indicate greater perceived safety of the HPV vaccine. A composite score was created by averaging the three items. Previous research has demonstrated acceptable reliability (Cronbach’s α = 0.86; Nan and Daily, [42]). The Cronbach alpha for the three items was equal to 0.90 in the current study.

#### Perceived harm

Three items that represent a subscale of the validated Carolina HPV Immunization Attitudes and Beliefs Scale (CHIAS) [38] assessed perceived harm. Sample items: “I think the HPV vaccine may cause health problems in the future”, “I think the HPV vaccine is unsafe”, and “I think the HPV vaccine might cause short-term problems like fever or discomfort.” Response options ranged from (1) Strongly disagree, to (5) Strongly agree. Higher scores indicate greater perceived harm. A composite score was created by averaging the three items. Previous studies have demonstrated high re-test reliability estimates (0.73—0.80; [15, 38]). The Cronbach alpha for the two items was equal to 0.70 in the current study.

#### Perceived severity

A single item adapted by Katz, Krieger, and Roberto [28] assessed the perceived severity of HPV infection for oneself: “How severe do you think genital HPV infection is for yourself?” Response options ranged from (1) Not at all severe, to (5) Extremely severe. A “not applicable” response option was provided and coded as missing.

#### Familism

An 18-item Familism scale from Lugo Steidel and Contreras [52] assessed familism. The scale has been administered in both English and Spanish in previous research with good internal reliability in Latino adults with low socioeconomic status. Sample item: “A person should rely on his or her family if the need arises.” Response options ranged from (1) Strongly disagree, to (5) Strongly agree. Previous research has demonstrated acceptable reliability for the familism scale (0.83; [52]. A composite score was created by averaging the 18 items. The Cronbach alpha for the familism scale was equal to 0.86 in the current study.

#### The Duke University Religion Index (DUREL)

The Duke University Religion Index (DUREL) is a five-item measure of religiosity. Three subscales are included in the DUREL to assess organizational religious activity, non-organizational religious activity, and intrinsic religiosity (Koenig and Büssing, [32]). Organizational religious activity is assessed by a single item: “How often do you attend church or other religious meetings?” Non-organizational activity is assessed by a single item: “How often do you spend time in private religious activities, such as prayer, meditation, or Bible study?” The intrinsic religiosity subscale is a 3-item scale that assesses personal religious commitment (Koenig and Büssing, [32]). Sample item: “In my life, I experience the presence of the Divine (i.e., God).” The intrinsic religiosity scale has demonstrated high internal consistency (Cronbach alphas = 0.78 – 0.91; Koenig and Büssing, [32]). The Cronbach alpha for the intrinsic religiosity subscale was equal to 0.85 in the current study.

#### Health-related community stigma

Two items assessed if individuals perceived health-related community stigma. Sample items: “People in your community will think less of a family or person with HPV.” “My community believes that health conditions can cause shame or embarrassment.” Response options ranged from (1) Strongly disagree, to (5) Strongly agree. Responses were reverse coded to range from (1) Strongly agree, to (5) Strongly disagree. A composite score was created by averaging the two items. Higher scores indicate higher perceived health-related community stigma. The Cronbach alpha for the two items was equal to 0.82 in the current study.

#### HPV informational sources

A single item assessed the number of HPV informational sources one has encountered: “If you have received information about the HPV vaccine, where were you given these recommendations or guidance on whether you should receive the HPV vaccine (select all that apply).” Fifteen response options were provided: “healthcare practitioner (e.g., pediatrician, family practice doctor)”, “a community health clinic”, “pharmacist”, “pharmacy”, “school nurse”, “family/friends”, “social media”, “Government website (e.g. CDC, FDA, etc.)”, “World Health Organization (WHO)”, “radio”, “television”, “internet”, “newspaper”, and “promotora”. An “other” option permitted text entry. Participants could select more than one response option and a composite was created by adding up responses coded as 1 = Yes and 0 = No. Total scores ranged from 0 to 14.

#### HPV trusted sources of information

An item assessing the frequency of using online sources or social media to obtain health information. Sample item: “If you were receiving information about HPV and the HPV vaccine, who would you believe is the most credible and trustworthy person would be to give you information? (select all that apply).” The same fifteen response options as the HPV informational source item above were provided. Participants could select more than one response option and a composite was created by adding up responses coded as 1 = Yes and 0 = No. Total scores could range from 0 to 14.

#### Government trust

A single item assessed trust in government. Sample item: “I would not get a vaccine because I do not trust what the government says about it.” Response options ranged from (1) Strongly disagree, to (5) Strongly agree. Responses were reverse coded to range from (1) Strongly agree, to (5) Strongly disagree. Higher scores indicate greater trust in the government.

### Dependent Variables

#### HPV Vaccine Uptake (HPV-VU)

A single item assessed if participants have received the HPV vaccine. Sample item: **“**Have you ever received the Human Papillomavirus (HPV) vaccine?” Response options included: 0 = No, 1 = Yes.

#### HPV Vaccine Acceptance (HPV-VA)

A single item assessed acceptance of the HPV vaccine. Sample item: “I intend to get vaccinated with the HPV vaccine.” Response options ranged from (1) Strongly disagree to (5) Strongly agree.

### Procedure

Participants were recruited through social media using Facebook geo-targeted advertisements and asked to complete a 30-min survey which included the above-described measures. Participants had the option of completing the survey in either English or Spanish and were provided with a $25 gift card for their participation. The survey was administered on QuestionPro software during June – August 2020. The Anti-Ballot Box Stuffing (ABBS) feature was enabled in Questionpro© to prevent multiple responses. The ABBS assigns a unique response ID to each respondent and saves cookies to their computers to track and prevent multiple responses.

### Approach to analysis

Descriptive statistics were calculated for demographic variables such as age, ethnicity, gender, and educational level. Percentages were examined for nominal variables; means and standard deviations were examined for continuous variables. Chi-square analyses examined relationships between categorical variables and correlational analyses examined relationships between continuous variables. For example, chi-square analyses examined the associations between vaccine uptake and demographic variables (e.g., gender, ethnicity, household income); correlation analyses examined the associations between vaccine acceptance and the independent variables (e.g., perceived safety, perceived benefits, perceived harm, and perceived severity).

A series of logistic and linear regression analyses were calculated to examine theory-based factors, culture-based factors, and trusted sources of information. Preliminary analyses were conducted for all regression analyses to ensure no serious violations of the assumptions of normality, linearity, multicollinearity, and homoscedasticity. Model 1: A logistic regression model examined theory-based predictors of HPV-VU. HPV-VU was entered as the dependent variable and the following variables were entered as independent variables: perceived safety, perceived benefits, perceived harm, and perceived severity were entered as the independent variables. Model 2: A linear regression model examined theory-based predictors of HPV-VA. HPV-VA was entered as the dependent variable and the following variables were entered as independent variables: perceived safety, perceived benefits, perceived harm, and perceived severity. Model 3: A logistic regression model examined culture-based predictors of HPV-VU. HPV-VU was entered as the dependent variable and the following variables were entered as independent variables: language, familism, religiosity (as indexed by the DUREL), household size, and health-related community stigma. Model 4: A linear regression model examined culture-based predictors of HPV-VA. HPV-VA was entered as the dependent variable and the following variables were entered as independent variables: language, familism, religiosity, household size, and health-related community stigma. Model 5: A logistic regression model examined trusted sources of information as predictors of HPV-VU. HPV-VU was entered as the dependent variable and the following variables were entered as independent variables: HPV informational sources, HPV trusted sources of information, and government trust. Model 6: A linear regression model examined trusted sources of information as predictors of HPV-VA. HPV-VA was entered as the dependent variable and the following variables were entered as independent variables: HPV informational sources, HPV trusted sources of information, and government trust.

## Results

Sixty-one percent (*n* = 367) of the sample completed the survey in English and 39% (*n* = 235) completed the survey in Spanish. Fifty-eight percent (*n* = 356) of the sample reported that Spanish was their primary language spoken at home. More than half (59.1%) of the sample reported that their annual income was below $40,000 and 72.6% of the sample reported to be parents (see Table 1).

**Table 1:** Demographic and background information (*N* = 602)

Variable	Total Responses	Percentage of Responses
Ethnicity		
Non-Hispanic	53	8.8%
Hispanic	534	88.7%
Prefer not to answer	10	1.7%
Missing	5	0.8%
Primary Language		
English	248	41.2%
Spanish	349	58%
Missing	5	0.8%
Language that survey was completed in		
English	367	61%
Spanish	235	39%
Household income		
Less than $5,000	64	10.6%
$5,001-$20,000	116	19.3%
$20,001-$40,000	176	29.2%
$40,001-$60,000	108	17.9%
$60,001-$80,000	73	12.1%
$80,001-$100,000	29	4.8%
$100,001 or more	17	2.8%
Do not know	8	1.3%
Other		
Prefer not to answer	8	1.3%
Missing	3	0.5%
Have you been sexually active at any point in your lifetime (e.g., oral sex, vaginal sex, anal sex)?		
Yes	533	88.5%
No	39	6.5%
Prefer not to answer	21	3.5%
Missing	9	1.5%
How often do you use protection (e.g., condom, vaginal condom) when you have sex?		
Never	144	23.9%
Rarely	91	15.1%
Usually	113	18.8%
Always	123	20.4%
Unsure	84	14.0%
Prefer not to answer	36	6.0%
Missing	11	1.8%
Have you ever received the Human Papillomavirus (HPV) Vaccine?		
No	392	65.1%
Yes	183	30.4%
I am not sure	20	3.3%
Missing	7	1.2%
Can you remember how many times you received the HPV vaccine?		
1 dose	43	7.1%
2 doses	41	6.8%
3 doses	57	9.5%
I do not remember	43	7.1%
Missing	418	69.4%
^a^If you were receiving information about HPV and the HPV vaccine, who would you believe is the most credible and trustworthy:		
Healthcare practitioner (e.g., pediatrician, family practice doctor)	528	87.7%
Community health clinic	187	31.1%
World Health Organization	141	23.4%
Government website (e.g., CDC, FDA, etc.,)	124	20.6%
Community health worker or promotor(a) de salud	95	15.8%
Pharmacist	80	13.3%
School nurse	71	11.8%
Family/friends	53	8.8%
Pharmacy (e.g., Walgreens, CVS, etc.,)	39	6.5%
Internet	39	6.5%
Television	27	4.5%
Social media	23	3.8%
Newspaper	17	2.8%
Radio	12	2.0%
Other	5	0.8%

^a^Indicates a variable which included a “select all that apply” response and thus does not total to 100%

Participants reported that they have been recommended the HPV vaccine from the following informational sources: a healthcare practitioner (e.g., pediatrician, family practice doctor) (60.1%), a community health clinic (16.4%), the internet (14.6%), family/friends (14.3%), television (9.8%), social media (9.6%), a school nurse (6.6%), community health worker or promotor(a) de salud (4.2%), a government website (e.g., CDC, FDA, etc.,) (3.5%), the World Health Organization (WHO) (3.5%), a pharmacist (2.8%), a pharmacy (2.2%), the radio (1.8%), the newspaper (1.3%), and “other” (5.1%). The most trusted sources of HPV information are provided in Table 1.

Approximately 65% of the sample reported that they have not received the HPV vaccine. Of those who reported having received the HPV vaccine, only 6.8% reported having received two doses, and 9.5% reported having received three doses (see Table 1). Nearly nine out of ten (88.5%) participants reported having been sexually active in their lifetime and nearly a quarter (23.9%) of the sample reported never using sexual protection (e.g., condoms). Approximately one out of five (18.3%) participants reported having had a sexually transmitted infection in their lifetime. A significant association emerged between having received the HPV vaccine, gender identity, and race (see Table 2).

**Table 2:** Comparison of demographics and HPV-VU (*N* = 602)

	HPV Vaccine Uptake			
Variable	No (n[%])	Yes (n[%])	Prefer not to answer (n[%])	*P-value*
Gender Identity				.001^a^
Male	71 (12)	20 (3.4)	9 (1.5)	
Female	308 (51.9)	159 (26.8)	10 (1.7)	
Gender Variant	7 (1.2)	3 (0.5)	0 (0)	
Prefer not to answer	3 (0.5)	1 (0.2)	0 (0)	
Other	1 (0.2)	0 (0)	1 (0.2)	
Race				.029^a^
White	277 (47.2)	130 (22.1)	11 (1.9)	
Black	7 (1.2)	5 (0.0)	0 (0)	
Asian	0 (0)	1 (0.2)	0 (0)	
Native-American	1 (1.0)	0 (0)	2 (2.1)	
Other	51 (8.7)	32 (5.5)	3 (0.5)	
Prefer not to answer	51 (8.7)	11 (1.8)	6 (1.0)	
Primary Language				.059
English	150 (25.3)	83 (14)	12 (2.0)	
Spanish	241 (40.6)	99 (16.7)	8 (1.3)	
Household income				.649^a^
Less than $5,000	42 (7.1)	18 (3.0)	4 (0.7)	
$5,001-$20,000	80 (13.4)	30 (5.0)	5 (0.8)	
$20,001-$40,000	111 (18.7)	58 (9.7)	5 (0.8)	
$40,001-$60,000	72 (12.1)	33 (5.5)	3 (0.5)	
$60,001-$80,000	48 (8.1)	23 (3.9)	1 (0.2)	
$80,001-$100,000	19 (3.2)	10 (1.7)	0 (0)	
$100,001 or more	13 (2.2)	4 (0.7)	0 (0)	
Do not know	3 (0.5)	4 (0.7)	1 (0.2)	
Prefer not to answer	4 (0.7)	3 (0.5)	1 (0.2)	

^a^Indicates that the ‘minimum expected cell frequency’ assumption of the chi-square test was violated and the *p*-value should be interpreted with caution

### HPV-VU and sexual experience

A chi-square test for independence indicated a significant association between HPV-VU and sexual activity in a lifetime, *Χ*^2^ (4, *n* = 591) = 109.93, *p* < 0.001, phi = 0.431. Specifically, of those who reported having sexual activity in their lifetime, 61.1% reported having not received the HPV vaccine compared to 26.9% who reported having received the HPV vaccine. Additionally, a chi-square test for independence indicated a significant association between having received the HPV vaccine and frequency of wearing sexual protection *Χ*^2^ (10, *n* = 589) = 33.92, *p* < 0.001, phi = 0.24. Specifically, of those who reported to never wear sexual protection, 17.7% reported to have not received the HPV vaccine compared to 6.5% who reported to have received the HPV vaccine.

### Personal and family vaccine beliefs

Correlational analyses revealed moderate to strong associations between personal and family vaccine beliefs, ranging from -0.34 to 0.75 (see Table 3). The strongest correlation was between the personal belief that vaccines cause autism and their family’s belief that vaccines cause autism (*r* = 0.75; *p* < 0.001).

**Table 3:** Personal and family beliefs about vaccines

	1	2	3	4	5	6	7	8	9	10
1. I had negative feelings about vaccines while I was growing up	–	–	–	–	–	–	–	–	–	–
2. I believe vaccines can cause serious illness	.53**	–	–	–	–	–	–	–	–	–
3. I believe vaccines are safe and effective	-.43**	-.54**	–	–	–	–	–	–	–	–
4. I believe everyone should be immunized against serious diseases	-.34**	-.49**	.65**	–	–	–	–	–	–	–
5. I believe vaccines cause autism	.45**	.59**	-.43**	-.43**	–	–	–	–	–	–
6. My family had negative feelings about vaccines while I was growing up	.50**	.49**	-.36**	-.35**	.49**	–	–	–	–	–
7. My family believes vaccines can cause serious illness	.52**	.59**	-.44**	-.37**	.60**	.73**	–	–	–	–
8. My family believes vaccines are safe and effective	-.43**	-.50**	.54**	.50**	-.51**	-.56**	-.65**	–	–	–
9. My family believes everyone should be immunized against serious diseases	-.41**	-.43**	.49**	.60**	-.52**	-.48**	-.52**	.65**	–	–
10. My family believes vaccines cause autism	.44**	.52**	-.44**	-.45**	.75**	.54**	.62**	-.60**	-.55**	–
Mean (SD)	2.07 (1.03)	2.24 (1.05)	3.94 (0.86)	4.15 (0.91)	1.93 (0.96)	1.93 (1.05)	1.92 (1.05)	4.00 (0.96)	4.11 (0.93)	1.86 (0.97)
N	570	570	570	570	571	571	571	571	570	569

Correlations are reported using Pearson’s Correlation Coefficient (2-tailed)

^*^p < *.05*

^**^p < *.01*

### Theory-based correlates of HPV-VA

Correlational analyses revealed that HPV-VA was associated with the following factors: 1) perceived safety, 2) perceived benefits 3) perceived harm, and 4) perceived severity. The correlations ranged from -0.37 to 0.30 (see Table 4).

**Table 4:** Correlates of HPV Vaccine Acceptance

	1	2	3	4	5	6	7
1. Age	–	–	–	–	–	–	–
2. HPV Knowledge	-.07	–	–	–	–	–	–
3. Perceived Benefits	-.04	.12**	–	–	–	–	–
4. Perceived Safety	-.03	.16**	.16**	–	–	–	–
5. Perceived Harm	-.03	-.08	-.20**	-.73**	–	–	–
6. Perceived Severity	-.01	-.10*	.10*	-.01	-.05	–	–
7. HPV-VA	-.12*	-.03	.29**	.25**	-.37**	.30**	–
Mean (SD)	34.65 (9.79)	68.33 (11.78)	3.81 (0.88)	2.94 (1.06)	2.74 (0.80)	3.04 (1.31)	3.28 (1.07)
N	542	541	585	589	589	581	571

*Note:* Correlations are reported using Pearson’s Correlation Coefficient (2-tailed)

^*^p < *.05*

^**^p < *.01*

### Theory-based predictors of HPV-VU

Direct logistic regression was performed to assess the impact of several factors on HPV-VU. The model contained four independent variables (perceived benefits, perceived safety, perceived harm, and perceived severity). The full model containing all predictors was statistically significant, *Χ*^2^ (4, *N* = 552) = 16.42, *p* = 0.003, indicating the model was able to distinguish between participants who reported and did not report having received the HPV vaccine. The model as a whole explained between 2.9% (Cox and Snell R square) and 4.1% (Nagelkerke R squared) of the variance in vaccine uptake, and correctly classified 68.3% of cases. As shown in Table 5, only one of the independent variables made a unique statistically significant contribution to the model (perceived safety). Increasing perceived safety was associated with an increased likelihood of HPV-VU.

**Table 5:** Logistic regression summary for theory-based factors predicting HPV-Vaccine Uptake (*N* = 552)

	*B*	S.E	Wald	*df*	*p*	Odds Ratio	95% CI for Odds Ratio Lower Upper	
Perceived Benefits	.07	.11	.42	1	.519	1.07	.87	1.32
Perceived Safety	.35	.13	6.99	1	.008	1.42	1.09	1.84
Perceived Harm	.02	.17	.01	1	.933	1.02	.72	1.43
Perceived Severity	.01	.07	.02	1	.904	1.01	.88	1.16
Constant	-2.15	.96	5.08	1	.024	.12		

Sample size is less than 602 due to missing data

### Theory-based predictors of HPV-VA

A multiple linear regression analysis was performed to predict HPV-VA based on perceived benefits, perceived safety, perceived harm, and perceived severity. The results indicate that perceived benefits (beta = 0.24,* t* = 4.20, *p* < 0.001), perceived harm (beta = -0.44,* t* = -4.92, *p* < 0.001), and perceived severity (beta = 0.22,* t* = 5.86, *p* < 0.001), significantly predicted HPV-VA, (*F* (4, 367) = 30.92, *p* < 0.001), with an *R*^2^ of 0.25. More specifically, for every one-unit change in perceived benefits, there is a 0.24 increase in HPV-VA, holding all other variables constant. In addition, for every one-unit change in perceived harm, there is a 0.44 decrease in HPV-VA, holding all other variables constant. Lastly, for every one-unit change in perceived severity, there is a 0.22 increase in HPV-VA, holding all other variables constant (see Table 6)*.*

**Table 6:** Linear regression summary for theory-based factors predicting HPV-Vaccine Acceptance (*N* = 368)

Variables	*B*	*SE B*	β	95% confidence intervals		*P*
				Lower	Upper	
Constant	2.99	.50		2.02	3.97	< .001
Perceived Benefits	.24	.06	.20	.13	.35	< .001
Perceived Safety	-.02	.07	-.02	-.16	.11	= .736
Perceived Harm	-.44	.09	-.33	-.62	-.27	< .001
Perceived Severity	.22	.04	.27	.15	.29	< .001

Sample size is less than 602 due to missing data

### Exploratory analyses

#### Culture and HPV-VU

Direct logistic regression was performed to explore the impact of cultural factors on HPV-VU. The model contained seven independent variables (primary language, household size [as a proxy of multi-generational households], familism, religiosity [3 subscales: intrinsic religiosity, organizational activity, and non-organizational activity], and health-related community stigma). The full model containing all predictors was statistically significant, *Χ*^2^ (7, *N* = 491) = 20.83, *p* = 0.004, indicating the model was able to distinguish between participants who reported and did not report having received the HPV vaccine. The model as a whole explained between 4.2% (Cox and Snell R square) and 5.8% (Nagelkerke R squared) of the variance in vaccine uptake, and correctly classified 67.8% of cases. As shown in Table 7, two of the independent variables made a unique statistically significant contribution to the model (household size and religiosity subscale: non-organizational activity). Increasing household size and non-organizational activity were associated with an increased likelihood of HPV-VU.

**Table 7:** Logistic regression summary for culture-based factors predicting HPV-Vaccine Uptake (*N* = 491)

	*B*	S.E	Wald	*df*	*p*	Odds Ratio	95% CI for Odds Ratio Lower Upper	
Primary Language	.17	.20	.71	1	.399	1.19	.80	1.77
Household Size	.15	.07	5.10	1	.024	1.16	1.02	1.33
Familism	.16	.18	.75	1	.388	1.17	.82	1.68
Intrinsic Religiosity	-.15	.11	1.89	1	.169	.86	.69	1.07
Organizational Activity	-.04	.09	.19	1	.660	.96	.82	1.14
Non-Organizational Activity	.14	.07	4.30	1	.038	1.15	1.01	1.31
Health-Related Community Stigma	.10	.09	1.09	1	.297	1.10	.92	1.33
Constant	-1.97	.93	4.48	1	.034	.14		

Sample size is less than 602 due to missing data

#### Culture and HPV-VA

A multiple linear regression analysis was performed to predict HPV-VA based on the seven cultural factors in the previous model (primary language, household size [as a proxy of multi-generational households], familism, religiosity [3 subscales: intrinsic religiosity, organizational activity, and non-organizational activity], and health-related community stigma). The results indicate that primary language (*β* = 0.30,* t* = 5.72, *p* < 0.001)*,* household size (*β* = 0.19,* t* = 3.74, *p* < 0.001)*,* organizational activity (*β* = 0.14,* t* = 2.09, *p* = 0.038)*,* and health-related community stigma (*β* = -0.13,* t* = -2.59, *p* = 0.01) significantly predicted HPV-VA, (*F* (7, 338) = 9.36, *p* < 0.001), with an *R*^2^ of 0.17 (see Table 8).

**Table 8:** Linear regression summary for culture-based factors predicting HPV-Vaccine Acceptance (*N* = 339)

Variables	*B*	*SE B*	β	95% confidence intervals		*P*
				Lower	Upper	
Constant	1.37	.56		.26	2.47	= .015
Primary Language	.65	.11	.30	.43	.87	< .001
Household Size	.14	.04	.19	.07	.21	< .001
Familism	.16	.10	.08	-.04	.36	= .119
Intrinsic Religiosity	.00	.06	.00	-.12	.12	= .99
Organizational Activity	.10	.05	.14	.01	.19	= .038
Non-Organizational Activity	-.02	.04	-.03	-.09	.06	= .67
Health-Related Community Stigma	-.13	.05	-.13	-.23	-.03	= .01

Sample size is less than 602 due to missing data

#### Informational sources, trust, and HPV-VU

Direct logistic regression was performed to explore the impact of HPV informational sources and trust on HPV-VU. The model contained three independent variables (HPV informational sources, HPV trusted sources of information, and government trust). The full model containing all predictors was statistically significant, *Χ*^2^ (3, *N* = 535) = 10.75, *p* = 0.013, indicating the model was able to distinguish between participants who reported and did not report having received the HPV vaccine. The model as a whole explained between 2% (Cox and Snell R square) and 2.8% (Nagelkerke R squared) of the variance in HPV-VU, and correctly classified 69.3% of cases. As shown in Table 9, only one of the independent variables made a unique statistically significant contribution to the model (HPV trusted sources of information). Increases in HPV trusted sources of information were associated with an increased likelihood of HPV-VU.

**Table 9:** Logistic regression summary for sources of information and trust predicting HPV-Vaccine Uptake (*N* = 535)

	*B*	S.E	Wald	*df*	*p*	Odds Ratio	95% CI for Odds Ratio Lower Upper	
HPV Informational Sources	.10	.08	1.51	1	.22	1.11	.94	1.30
HPV Trusted Sources of Information	.11	.05	5.41	1	.02	1.12	1.02	1.23
Government Trust	.06	.09	.39	1	.54	1.06	.88	1.28
Constant	-1.47	.40	13.27	1	.000	.23		

Sample size is less than 602 due to missing data

#### Informational sources, trust, and HPV-VA 

A multiple linear regression analysis was performed to predict HPV-VA from HPV informational sources and trust. The model contained three independent variables (HPV informational sources, HPV trusted sources of information, and government trust). As shown in Table 10, government trust (*β* = 0.31,* t* = 6.19, *p* < 0.001) significantly predicted HPV-VA, (*F* (3, 358) = 13.92, *p* < 0.001), with an *R*^2^ of 0.11.

**Table 10:** Linear regression summary for sources of information and trust predicting HPV-Vaccine Acceptance (*N* = 370)

Variables	*B*	*SE B*	β	95% confidence intervals		*P*
				Lower	Upper	
Constant	1.90	.23		1.45	2.34	< .001
HPV Informational Sources	.03	.05	.03	-.07	.13	= .609
HPV Trusted Sources of Information	.03	.03	.06	-.03	.09	= .258
Government Trust	.33	.05	.31	.22	.43	< .001

Sample size is less than 602 due to missing data

#### Gender and ethnicity

There was an overrepresentation of individuals who identified as female (79.6%) and who were Hispanic (89.4%). To account for this overrepresentation, additional analyses were conducted to explore the influence of gender and ethnicity on HPV-VA and HPV-VU. For statistical purposes, gender was dichotomized and coded as females = 0 and males = 1. Similarly, ethnicity was dichotomized and coded as non-Hispanic = 0 and Hispanic = 1. A multiple linear regression analysis was performed to predict HPV-VA based on gender and ethnicity. The results indicate that ethnicity (*β* = -0.18, *t* = -3.53, *p* < 0.001) significantly predicted HPV-VA, (*F* (2, 358) = 6.66, *p* < 0.001), with an R^2^ of 0.04. Specifically, non-Hispanics were more likely to accept the HPV vaccine than Hispanics. There were no differences in individuals who identified as male or female. To further explore gender, a follow-up Mann–Whitney *U* test was conducted and also revealed no significant difference in HPV-VA between females (*Md* = 3.0, *n* = 289) and males (*Md* = 3.0, *n* = 70), *U* = 9423.50, *z* = -0.926, *p* = 0.355. Direct logistic regression was performed to explore the impact of gender and ethnicity on HPV-VU. The full model containing all predictors was statistically significant, *Χ*^2^ (2, *N* = 550) = 6.932, *p* = 0.031, indicating the model was able to distinguish between participants who reported and did not report having received the HPV vaccine. The model as a whole explained between 1.3% (Cox and Snell R square) and 1.8% (Nagelkerke R squared) of the variance in HPV-VU, and correctly classified 67.8% of cases. Gender made a unique statistically significant contribution to the model (*β* = -0.60, *p* = 0.029). Specifically, HPV-VU was higher in females than males. There were no differences in HPV-VU between Hispanics and non-Hispanics. To further explore ethnicity (Hispanics and non-Hispanics), a follow-up Chi-square test for independence (with Yates Continuity Correction) was conducted and also revealed no significant association between HPV-VU and ethnicity, χ^2^ (1, *n* = 558) = 0.80, *p* = 0.372, phi =—0.044).

## Discussion

The current study highlights both previously assumed and novel findings exploring vaccination behaviors among a predominately Hispanic, vaccine-eligible (ages 18–45 years old) adult population. Our hypothesis that theory-based factors would be associated with HPV-VA was supported. Specifically, correlation analyses revealed that HPV-VA was negatively associated with perceived harm and positively associated with perceived benefits, perceived safety, and perceived severity. These correlations are consistent with prior research (Brewer & Fazekas, [7]; [45]), suggesting that despite theory-based factors commonly being assessed in Caucasian samples, these factors are seemingly generalizable to other ethnicities/races including Hispanics. A study by Sledge [51] determined that perceived severity, perceived benefit, perceived barriers, and self-efficacy significantly predicted young college-aged males’ intention to receive the HPV vaccination (*R*^2^ = 0.526; *F*_5,57_ = 12.636, *p* < 0.001). Although theory-based factors have demonstrated generalizability, other factors should continue to be explored (i.e., local norms) to fully understand the depth and breadth of vaccine uptake and acceptance in vulnerable populations (i.e., Hispanics).

The strongest association in the current study was between family-held beliefs that vaccines cause autism and one’s own belief that vaccines cause autism. This finding may be related to fraudulent research by Andrew Wakefield which suggested that the MMR vaccine caused autism (see Motta and Stecula, [40] for more information). We believe that family-focused health communications and interventions that effectively dispel myths and misperceptions about the HPV vaccine may be warranted. Another important finding in the current study is that there was not a significant association observed between knowledge about HPV and HPV-VA. Although counter-intuitive, this finding is consistent with prior studies that also reported no effect [14, 19]. Collectively, we believe that these findings may inform the development of future interventions by highlighting that increasing knowledge about HPV may make individuals more knowledgeable, but likely does not translate into increases in HPV-VA or HPV-VU. When designing interventions researchers should have primary goals of increasing other factors aside from knowledge (e.g., increasing perceived benefits and safety of the HPV vaccine) to have a greater impact on HPV-VA and HPV-VU.

Importantly, we explored how trust in the government and various sources of information might influence HPV-VA and HPV-VU. Trust is a critical factor in healthcare and the formation and maintenance of productive relationships between patients and providers. Medical mistrust can prevent individuals from seeking healthcare and limit compliance with medical advice or treatment. Specific to HPV-VA and HPV-VU, provider recommendation has been established as one of the most important variables in the decision to vaccinate [49]. Trust in a provider, coupled with strong recommendation behavior, should result in higher vaccination rates and reduced cancer incidence over time. Indeed, parents in the U.S. [41] and mothers in England [37] demonstrated higher HPV-VA and increased likelihood of vaccinating their children when they were more trusting of medical authorities. Moreover, members of racial and ethnic minority groups in the U.S. tend to be highly trusting of doctors and other healthcare providers [21], although differences are observed among these groups. Bahena et al. [4] reported that Mexican mothers living in the U.S. or Mexico have increased HPV-VA and are willing to vaccinate their children if information about the vaccine and HPV comes from a medical provider. Other studies have reported high levels of trust in physicians and higher willingness to vaccinate in response to provider recommendations among Hispanic, Somali, and Ethiopian/Eritrean parents [20] as well as young African American, Latina, Haitian and Caucasian women of low income [26]. Interestingly, Latinx fathers demonstrate high levels of trust in healthcare providers and increased HPV-VA if a provider recommendation is given (Lindsay et al., [35]), but a separate investigation showed that Hispanic men are less willing than other ethnicities to trust information from doctors about cancer (Cooper et al., [13]). It is also reported that HPV-VA increases among both Latina [22] and Black [30] women with high levels of medical mistrust if the recommending provider is female and/or of the same race or ethnicity as the patient.

Historical and current factors lead to increased mistrust of medicine, the government, and other sources of healthcare information among patients. This is especially true for Blacks and African Americans, as a consequence of the exploitation and human rights violations that occurred in the Tuskegee syphilis study and continue to impact their medical and research participation [1, 50]. More recently, health disparities and socioeconomic influences on health have led to increasing mistrust among diverse groups including Blacks, Latina immigrants, rural Latinx populations, and transgender individuals [25]. While trust in government healthcare agencies tends to be high among patient populations, trust in the federal government as a whole varies and has been reduced in some groups by the recent SARS-CoV-2 pandemic [12, 16] reported that Blacks displayed the most hesitancy toward receiving the COVID-19 vaccine, and this hesitancy declined the slowest over time. In another study, COVID-19 vaccine acceptance among Whites was greater than among Blacks or Hispanics one year into the pandemic [33]. While Blacks and Hispanics in this study were equally likely to refuse vaccination, the authors found that Hispanic participants indicated a higher willingness to vaccinate which suggests that tailored interventions may be effective in increasing vaccine uptake in specific groups. Prior research from our group suggests that younger adults were less likely to trust government-recommended vaccines compared to older adults [2] and individuals with high levels of government distrust also have reduced uptake of the flu vaccine [47]. Trust in online or other media sources of health information also varies according to several factors including the prior experience of the user and the reputation of the organization sponsoring the website or material [25, 29]. In these studies, higher levels of trust were associated with being younger, more educated, female, Caucasian, in good health, and having a higher income. Trust in websites increased further if they were easy to navigate, responded quickly, and had an attractive design and appearance. In contrast, another study reported that minority group members were more likely than Caucasians to trust health information from media sources [21], with non-Hispanic Blacks also showing increased trust in information from charitable organizations and religious organizations or leaders [24].

Several important findings emerged related to the influence of Hispanic culture on HPV-VA and HPV-VU. Primary language*,* household size*,* organizational religious activity, and health-related community stigma were statistically significant predictors of HPV-VA. Specifically, individuals were more likely to accept the HPV vaccine if Spanish was the primary language at home. Furthermore, household size was positively associated with HPV-VA, suggesting that individuals residing in larger households were more likely to accept the HPV vaccine. Organizational religious activity was positively associated with HPV-VA, suggesting that individuals were more likely to accept the HPV vaccine if they reported higher frequency of attending church or other religious meetings. Health-related community stigma was inversely associated with HPV-VA, suggesting that individuals are less likely to accept the HPV vaccine if they experience higher levels of stigma from their community. Lastly, nonorganizational religious activity was positively associated with HPV-VU, suggesting that individuals were more likely to accept the HPV vaccine if they spent more time participating in private religious activities, such as prayer, meditation, or Bible study. Importantly, few or no studies have examined specific culture-based factors in predominately Hispanic samples. We believe there is a critical need for identifying numerous culture-based factors associated with vaccine acceptance in Hispanics to inform the development of culturally tailored interventions for promoting the HPV vaccine. Hispanic mothers have been previously known for placing their own needs on hold to put the health and wellbeing of their children first, to ensure that they are healthy and protected (Aragones et al., [3]); therefore, it is critical to investigate how Hispanic parents’ perceptions influence vaccine rates in their children. Lindsay and colleagues [36] identified that there was a high mother-to-daughter acceptance rate of HPV vaccination with consensus about the health benefits conferring protection against HPV infection and the inherent risk of cervical cancer. Although most participants’ daughters had a high HPV vaccine initiation rate, many were unaware whether their daughters had completed the vaccine series [36]. In a separate study, Lindsay and colleagues [34] also identified that mothers were aware of and believed in the importance of the HPV vaccine. Although there may be high acceptance and/or consensus rates on HPV vaccination, Hispanic parents still are influenced by social-cultural constructs. For example, Lindsay et al. [36] identified hesitation on behalf of parents when speaking to their children about HPV and the HPV vaccine, as they feared such conversations would lead to uncomfortable and tabooed sexuality and sexual health discussions. Fernandez-Pineda and colleagues [18] also identified sex myths among parents to be linked to HPV vaccination and taboo, while also identifying cultural constructs of *machismo* and *marianismo*, as deterrents and influencers of parent perceptions of HPV vaccines. Although most of the available literature is performed on Hispanic mothers, Kornfeld and colleagues [31] found that Hispanic fathers (predominantly Mexican) reported positive attitudes and a high willingness to vaccinate both daughters and sons with the HPV vaccine.

Lastly, it is important to note that more than half of our sample reported not having received the HPV vaccine. This finding was unexpected given that El Paso County has been estimated to have the highest up-to-date HPV vaccination rates in Texas [44]. The low HPV-VU rates in the current study might be explained by the average age of our sample being approximately thirty-five. Prior to 2018, HPV vaccine was only recommended for individuals below the age 27. The U.S. Food and Drug Administration expanded the use of the HPV vaccine for adults between the ages 27—45 years in late 2018 [54]. It could be that many older adults in the study were not eligible for the vaccine given their age or they simply did not feel the need to get vaccinated given their age. Future studies should explore age-related differences in HPV vaccination rates in this population.

### Implications

With the rapidly growing Hispanic population and increased diversity, healthcare professionals and practitioners should be prepared to identify and address potential barriers and challenges associated with HPV cancer prevention efforts. The importance of this subject matter cannot be overly emphasized. Accessibility of vaccines and health care services is a complex and multidimensional construct that seeks to capture a complex situation that involves health-seeking values, expectations, and patterns as well as organizational factors that can facilitate or hinder receiving HPV quality services. The changing demographics of Hispanics means that organizations and providers need to be continually attentive to how the Hispanic community is changing, meaning that service delivery must be culturally responsive to be viable and relevant. Implications in the areas of practice, policy, and research are provided.

### Practice

Findings from this study support well-established data of utilizing theory-based frameworks (i.e., the Health Belief Model) to inform the development of future vaccine acceptance/uptake interventions regardless of ethnicity/race. Utilizing both theory-based and exploratory cultural data could offer opportunities for future education models that help increase vaccine uptake and acceptance within a specific ethnicity/race (e.g., Hispanic). In addition, the data collected may assist in addressing any vaccine misinformation and hesitancy in light of challenges as they related to COVID-19 and its impact on vaccine confidence. Additionally, the incorporation of data based on exploratory cultural constructs should be considered when developing interventions for HPV-VU (i.e., increasing household size, non-organizational activity) and HPV-VA (i.e., primary language, increasing household size, organizational activity). Educational interventions tailored for communities can help address HPV-VU and HPV-VA.

### Policy

Understanding community needs, attitudes, and perceptions can help inform strategic interventions that address vaccine hesitancy and misinformation. Every community has different needs and challenges as they relate to vaccine uptake and acceptance. Utilizing the findings of this study to create interventions grounded in theory-based frameworks allows for opportunities for advocacy at both local and state levels. A variety of platforms (i.e., social media, print) can be used that are cost-efficient and simple to execute.

### Research

Theory-based interventions (i.e., the Health Belief Model) have demonstrated success in increasing vaccine uptake and acceptance. In vulnerable communities, additional research is needed that incorporates other factors and considerations including understanding discrimination and its roles in health behaviors, how the individual feels about their community (i.e., job availability, crime, parks, air population), and its role in health behaviors, and mental health.

### Limitations

The research design has some key limitations. The survey was implemented at a single point in time, offering a snapshot of theory-based and culture-based correlates of HPV-VA and HPV-VU. The cross-sectional nature of the data creates limited leverage in estimating causal determinants. In addition, the survey was offered online, which allowed participants to self-select into the survey. It could also be the case that unobservable factors influenced decisions about whether to participate in the survey. For example, providing a $25.00 gift card for participation may have influenced decisions to participate in the study and lower income individuals may have been persuaded to participate. Our study also failed to assess whether participants had medical insurance or affiliations to health services, which could have yielded an understanding of the barriers toward vaccination in our community. Another limitation is that the measures were self-reported by respondents, which may contribute to social desirability bias [43]. The online and confidential response mode likely reduces the threat of social desirability bias, however, it does not eliminate the threat. Lastly, our sample consisted primarily of Hispanic participants and who identified predominately as female. Given the overrepresentation of respondents who reported to be Hispanic and female, conclusions about differences in HPV-VA and HPV-VU could not be made by ethnicity or gender.

## Conclusion

Understanding vulnerable communities’ perceptions and attitudes towards vaccines inform the development of education information. Adequate HPV vaccination uptake among all vaccine-eligible populations is an important step to lessen the HPV-attributed cancer burden. Several other important findings emerged from the current study highlighting the need for health professionals and practitioners to tailor interventions to Hispanics embracing cultural and language preferences. Family is a key socialization mechanism in shaping Hispanic health-seeking behaviors. Integrating the cultural values of familism, personalism, collectivism, and marianismo/machismo into social and health interventions will help increase the likelihood of participation and meaningful engagement [17].

## Data Availability

The data generated and analyzed during the current study are publicly available at: https://data.mendeley.com/datasets/rg58w44khr

## Abbreviations

HPV: Human Papillomavirus
STI: Sexually transmitted infection
HPV-VU: Human papillomavirus vaccine uptake
HPV-VA: Human papillomavirus vaccine acceptance
HBM: Health Belief Model
